# Goal Attainment in Managing Pain and Depressive Symptoms Among Middle-Aged and Older Women

**DOI:** 10.1093/geroni/igaf122.3375

**Published:** 2025-12-31

**Authors:** Fatmata Barrie, Catherine Clair, Shelbie Atkins, Sarah Szanton, Janiece Taylor

**Affiliations:** Johns Hopkins University, Burtonsville, Maryland, United States; Johns Hopkins School of Nursing, Baltimore, Maryland, United States; Johns Hopkins University, Baltimore, Maryland, United States; Johns Hopkins University, Baltimore, Maryland, United States; Johns Hopkins University, Baltimore, Maryland, United States

## Abstract

Goal setting is a key component of pain interventions, fostering self-efficacy and engagement while addressing individualized pain and emotional well-being needs. While goal setting is widely recognized as beneficial, less is known about how goal priorities vary by intervention, population characteristics, and lived context. This study examines goal-setting and attainment in two populations: (1) Middle-aged and older African American women who were frail or prefrail in the DAPPER (Depression and Pain Perseverance through Empowered Recovery) study (N = 34) and (2) Middle-aged and older women of any race/ethnicity with mobility disabilities in the Women in PRIME (Pain Reduction and Improved Mood through Empowerment) study (N = 9). This analysis categorizes and compares 75 total goals set across these interventions to examine factors that influence self-management priorities. A comparative analysis of goals and goal domains revealed distinct priorities between the two groups. Goals were categorized into physical activity, pain management, emotional well-being, stress reduction, and social engagement. DAPPER participants prioritized physical activity goals (27.5%), likely influenced by the COVID-19 pandemic, which limited mobility. PRIME participants set broader goals, emphasizing social engagement, stress reduction, and emotional well-being, aligning with post-pandemic recovery. Differences in living situations and social networks may have also influenced goal-setting behaviors. Findings suggest that participants’ lived experiences, including temporality and social supports, shape goal-setting behaviors. These results highlight the need for individualized, patient-directed approaches that consider social determinants of health and cultural influences. Future research with larger samples is needed to confirm these patterns and optimize behavioral interventions for diverse populations.

